# Cancer Risk in Patients With Muscular Dystrophy and Myotonic Dystrophy: A Register-Based Cohort Study

**DOI:** 10.1212/WNL.0000000000209883

**Published:** 2024-09-19

**Authors:** Carolina Maya-González, Giorgio Tettamanti, Fulya Taylan, Anna Skarin Nordenvall, Thomas Sejersen, Ann Nordgren

**Affiliations:** From the Department of Molecular Medicine and Surgery, Center for Molecular Medicine (C.M.G., G.T., F.T., A.S.N., A.N.), Unit of Epidemiology, Institute of Environmental Medicine (G.T.), and Department of Women's and Children's Health (T.S.), Karolinska Institutet; Department of Clinical Genetics and Genomics (F.T., A.N.), Department of Radiology (A.S.N.), and Department of Child Neurology, Astrid Lindgren Children's Hospital (T.S.), Karolinska University Hospital, Stockholm; Department of Clinical Genetics and Genomics (A.N.), Sahlgrenska University Hospital, Gothenburg; and Institute of Biomedicine, Department of Laboratory Medicine (A.N.), University of Gothenburg, Sweden.

## Abstract

**Background and Objectives:**

Muscular dystrophies and myotonic disorders are genetic disorders characterized by progressive skeletal muscle degeneration and weakness. Epidemiologic studies have found an increased cancer risk in myotonic dystrophy, although the cancer risk spectrum is poorly characterized. In patients with muscular dystrophy, the cancer risk is uncertain. We aimed to determine the overall cancer risk and cancer risk spectrum in patients with muscular dystrophy and myotonic dystrophy using data from the Swedish National registers.

**Methods:**

We performed a matched cohort study in all patients with muscular dystrophy or myotonic dystrophy born in Sweden 1950–2017 and 50 matched comparisons by sex, year of birth, and birth county per individual. The association with cancer overall and specific malignancies was estimated using stratified Cox proportional hazard models.

**Results:**

We identified 2,355 and 1,968 individuals with muscular dystrophy and myotonic dystrophy, respectively. No increased overall cancer risk was found in muscular dystrophy. However, we observed an increased risk of astrocytomas and other gliomas during childhood (hazard ratio [HR] 8.70, 95% CI 3.57–21.20) and nonthyroid endocrine cancer (HR 2.35, 95% CI 1.03–5.34) and pancreatic cancer (HR 4.33, 95% CI 1.55–12.11) in adulthood. In myotonic dystrophy, we found an increased risk of pediatric brain tumors (HR 3.23, 95% CI 1.16–9.01) and an increased overall cancer risk in adults (HR 2.26, CI 1.92.2.66), specifically brain tumors (HR 10.44, 95% CI 7.30–14.95), thyroid (HR 3.92, 95% CI 1.70–9.03), and nonthyroid endocrine cancer (HR 7.49, 95% CI 4.47–12.56), endometrial (HR 8.32, 95% CI 4.22–16.40), ovarian (HR 4.00, 95% CI 1.60–10.01), and nonmelanoma skin cancer (HR 3.27, 95% CI 1.32–8.13).

**Discussion:**

Here, we analyze the cancer risk spectrum of patients with muscular dystrophy and myotonic dystrophy. To the best of our knowledge, this is the first report of an increased risk for CNS tumors in childhood and adult nonthyroid endocrine and pancreatic cancer in muscular dystrophy. Furthermore, for myotonic dystrophy, we confirmed previously reported associations with cancer and expanded the cancer spectrum, finding an unreported increased risk for nonthyroid endocrine cancer. Additional studies confirming the cancer risk and delineating the cancer spectrum in different genetic subtypes of muscular dystrophies are warranted before considering altered cancer screening recommendations than for the general population.

## Introduction

Muscular dystrophies are a group of genetic disorders with a prevalence between 3.8 and 26.8 in 100,000 individuals in different populations,^1^ characterized by progressive skeletal muscle degeneration and weakness. There are more than 40 genes associated with muscular dystrophy, resulting in diseases with varying degrees of severity, patterns of affected muscles, and age at onset. The presence of systemic involvement is also disease dependent.^2^

The most common muscular dystrophy diagnosed in children is Duchenne muscular dystrophy (DMD, MIM #310200), an X-linked disorder caused by pathogenic variants in the *DMD* gene, with a prevalence of 19 per 100,000 live male births in Sweden.^3^ Variants in the *DMD* gene leading to partially functional protein result in a milder disease phenotype known as Becker muscular dystrophy^4^ (BMD, MIM #300376). Although BMD has a more benign presentation, both diseases are characterized by progressive symmetric muscle weakness, with a more prominent proximal than distal impact, and often calf hypertrophy.^5^ By teen years, most patients with DMD and many with BMD require the use of a wheelchair. Respiratory insufficiency and cardiomyopathy usually develop as the disease progresses and are common causes of death in these patients.^5^

Myotonic dystrophies (DM1 and DM2, MIM #160900 and #602668, respectively) are autosomal dominant diseases caused by repeat expansions of the *DMPK* and *CNBP* genes in DM1 and DM2, respectively. The molecular mechanism includes nuclear aggregation of the proteins. These aggregates sequester regulators of protein-splicing, leading to mis-splicing of multiple downstream effector genes, accounting for the multisystemic involvement of both diseases.^6^ In the literature, myotonic dystrophy is classified as a muscular dystrophy. However, muscular dystrophies and myotonic dystrophies are 2 different muscular disorders; with myotonic dystrophy characterized by myotonia, among other symptoms.

DM1 has an estimated prevalence of 0.4–36.3 in 100,000 individuals, widely variable across populations,^7^ and it is characterized by distal muscle weakness, myotonia, and cataract. Severe cases also present with respiratory deficit, cardiac arrhythmia, intellectual disability, neuropsychiatric problems, and gastrointestinal and endocrine systemic involvement, among others.^8^ DM2 is likely underdiagnosed but has an estimated prevalence of 0.0–24.0 in 100,000 individuals, highly depending on the geographical areas in the world.^7^ Its clinical presentation usually has an onset in adulthood and includes muscle weakness, myotonia, and cataract. Severe forms include cardiomyopathy, insulin insensitivity, endocrine dysfunction, gastrointestinal involvement, and hearing impairment, among others.^9^ In DM1, but not in DM2, disease severity correlates with the size of the repeat expansion.^8,9^

Mouse models of multiple muscular dystrophies have a high incidence of rare muscular soft tissue sarcomas.^10-13^ However, no population studies on cancer risk in patients with muscular dystrophy have been conducted. Therefore, the cancer incidence in patients with muscular dystrophy remains unknown. Conversely, multiple epidemiologic studies of cancer in patients with myotonic dystrophy have been performed.

A register-based study using data from Sweden and Denmark between 1977 and 2008 found an overall cancer risk increase in patients with myotonic dystrophy, specifically of brain, endometrial, ovarian, colon, and pancreatic cancer.^14^ The increased risk of brain tumors was also reported in a subsequent Swedish register-based study that investigated the link between brain neoplasms and myotonic dystrophy using data from the Swedish National Registers.^15^ Furthermore, a US study observed an increased risk of choroidal melanoma and thyroid cancer, as well as indications of increased risk of testicular and prostate cancer in individuals with myotonic dystrophy.^16^ A second US study found an overall cancer risk increase in individuals with myotonic dystrophy, with the strongest associations observed for endometrial and testicular cancer and non-Hodgkin lymphoma.^17^ Additional studies in the United Kingdom also found an overall cancer risk increase in patients with myotonic dystrophy, driven by thyroid, uterus, and skin cancer; basal cell carcinoma; and cutaneous melanoma.^18,19^ Finally, a Spanish study observed an overall increased cancer risk in patients with myotonic dystrophy, particularly for cancer of the thyroid, brain, ovary, and endometrium.^20^

The aim of the current study was to use data from different national health registers in Sweden to investigate the overall cancer risk and risk of specific malignancies in individuals with muscular dystrophy and myotonic dystrophy.

## Methods

### Data Description

Thanks to the unique personal identification number assigned to Swedish residents,^21^ we have identified all individuals born in Sweden between 1950 and 2017 who had a diagnosis of muscular dystrophy or myotonic dystrophy in the National Patient Register (NPR)^22^ (*ICD-10* = G71.0, *ICD-9* = 359B for muscular dystrophy; *ICD-10* = G71.1, *ICD-9* = 359C for myotonic dystrophy). The NPR has hospital discharge diagnosis from 1964, and its coverage has increased with time, becoming nationwide in 1987. Information regarding outpatient specialist care diagnoses is available from 2001.

As some individuals had both muscular dystrophy and myotonic dystrophy diagnoses in the NPR, we classified them in the following way: (1) If they had a diagnosis of BMD or DMD, they were classified as having muscular dystrophy, regardless of the number of myotonic dystrophy diagnoses; (2) if they had at least 2 diagnoses of myotonic dystrophy, they were classified as having myotonic dystrophy, regardless of the number of muscular dystrophy diagnoses; (3) if they had only 1 diagnosis of myotonic dystrophy and at least 2 diagnoses of muscular dystrophy, they were classified as having muscular dystrophy; and (4) if they had 1 diagnosis of muscular dystrophy and 1 of myotonic dystrophy, we used the most recent diagnosis.

After identifying all patients with muscular dystrophy and myotonic dystrophy, we randomly selected individuals from the general population, who never had a diagnosis of muscular dystrophy or myotonic dystrophy in the NPR, using data from the Total Population Register.^23^ Individuals with muscular dystrophy or myotonic dystrophy were matched to 50 comparisons by sex, year of birth, and county of residence at birth. Separate matched cohorts were created for individuals with muscular dystrophy and myotonic dystrophy. We then linked our matched cohorts to the National Cancer Register,^24^ which covers the entire Swedish population and was established in 1958, to retrieve information on cancer diagnoses.

The individuals in our matched cohorts were followed from birth until the earliest of the following events: first cancer diagnosis, first emigration, death, or end of study period (December 31, 2017). Information regarding dates of emigration and death were obtained from the Total Population Register.^23^

Stratified Cox proportional hazard models were used to evaluate the association between muscular dystrophy or myotonic dystrophy and cancer. Separate analyses were performed for both diseases. Benign tumors or cancer in situ were not considered when studying cancer risk, except for benign CNS tumors. Separate analyses were performed in children (younger than 20 years) and adults (20 years or older). We first assessed the overall cancer risk (i.e., all cancer diagnoses combined) and then evaluated the risk of specific malignancies. In a sensitivity analysis, we excluded all individuals in our matched cohort diagnosed with a genetic syndrome in the NPR or in the Medical Birth Register,^25^ which contains birth characteristics and perinatal diagnoses on nearly all children born in Sweden from 1973. When studying the cancer risk in individuals with muscular dystrophy, we performed an additional sensitivity analysis, excluding patients with a BMD or DMD diagnosis.

All analyses were inherently adjusted for sex, year of birth, and county of residence at birth. When studying childhood cancer, we additionally adjusted for maternal education and parental age because associations between both parental age^26,27^ and socioeconomic factors^28^ and childhood cancer risk have been reported. We also performed sex-stratified analyses to evaluate whether the cancer risk associations in muscular dystrophy and myotonic dystrophy differed between men and women, including sensitivity analyses excluding patients with X-linked disorders, that is, BMD and DMD diagnoses.

The results were reported as hazard ratios (HRs) with 95% CIs. To avoid unstable risk estimates, we performed the analyses only if there were at least 3 individuals with muscular dystrophy/myotonic dystrophy who had the specific cancer diagnosis of interest. Data preparation was done using SAS software version 9.4, and statistical analyses were performed using Stata software version 16.1.

### Standard Protocol Approvals, Registrations, and Patient Consents

This study was approved by the Regional Ethical Review Board in Stockholm (Dnr 2018/1849-32).

### Data Availability

Pseudonymized personal data were obtained from the national Swedish Registry holders after ethical approval and secrecy assessment. According to Swedish laws and regulations, personal sensitive data can only be made available to researchers who fulfil legal requirements for access. Contact Professor Ann Nordgren (ann.nordgren@ki.se) for questions about data access.

## Results

We identified a total of 4,323 individuals born 1950 onwards who had a diagnosis of muscular dystrophy or myotonic dystrophy in the NPR. Approximately 18% of them (n = 790) had both a diagnosis of muscular dystrophy and myotonic dystrophy. After applying the criteria described in the methods section, we singled out 2,355 and 1,968 individuals with muscular dystrophy and myotonic dystrophy, respectively. The descriptive characteristics of the 2 matched cohorts are reported in Table 1.

**Table 1:** Characteristics of the Study Population

	Muscular dystrophy (n = 2,355)	Matched comparisons (n = 117,750)
Sex		
Men	1,472 (62.5)	73,600 (62.5)
Women	883 (37.5)	44,150 (37.5)
Birth year		
1950–1959	447 (19.0)	22,350 (19.0)
1960–1969	426 (18.1)	21,300 (18.1)
1970–1979	354 (15.0)	17,700 (15.0)
1980–1989	366 (15.5)	18,300 (15.5)
1990–1999	384 (16.3)	19,200 (16.3)
2000–2009	261 (11.1)	13,050 (11.1)
2010–2017	117 (5.0)	5,850 (5.0)
Maternal education		
Low	712 (30.2)	30,801 (26.2)
Medium	937 (39.8)	47,004 (39.9)
High	603 (25.6)	3,423 (29.2)
Missing	103 (4.4)	5,522 (4.7)
Maternal age^a^	27 (24–32)	27 (24–32)
Paternal age^a^	30 (26–35)	30 (26–35)
Genetic syndrome	31 (1.3)	426 (0.4)

	Myotonic dystrophy (n = 1,968)	Matched comparisons (n = 98,400)
Sex		
Men	990 (50.3)	49,500 (50.3)
Women	978 (49.7)	48,900 (49.7)
Birth year		
1950–1959	415 (21.1)	20,750 (21.1)
1960–1969	442 (22.5)	22,100 (22.5)
1970–1979	384 (19.5)	19,200 (19.5)
1980–1989	301 (15.3)	15,050 (15.3)
1990–1999	241 (12.3)	12,050 (12.3)
2000–2009	140 (7.1)	7,000 (7.1)
2010–2017	45 (2.3)	2,250 (2.3)
Maternal education		
Low	592 (30.1)	28,828 (29.3)
Medium	821 (41.7)	39,266 (39.9)
High	449 (22.8)	25,193 (25.6)
Missing	106 (5.4)	5,113 (5.2)
Maternal age^a^	27 (23–31)	27 (23–31)
Paternal age^a^	30 (26–35)	30 (26–34)
Genetic syndrome	41 (2.1)	330 (0.3)

a Median age (interquartile range).

While more than 60% of individuals with muscular dystrophy were male, no sex difference was observed among individuals with myotonic dystrophy. The number of individuals with muscular dystrophy and myotonic dystrophy decreased by birth year. Maternal education was lower among individuals with muscular dystrophy and myotonic dystrophy, particularly muscular dystrophy; while parental age was very similar in individuals with muscular dystrophy, myotonic dystrophy, and their matched comparisons. Moreover, the occurrence of other genetic syndromes was higher among individuals with muscular dystrophy and myotonic dystrophy (1.3% and 2.1%, respectively) than among the matched comparisons (0.3%–0.4%).

The results regarding the cancer risk in individuals with muscular dystrophy and myotonic dystrophy are reported in Tables 2 and 3, respectively. No increased overall cancer risk during childhood or adulthood was found among individuals with muscular dystrophy (Table 2). While there were only 8 pediatric cancer cases, 6 of them were CNS tumors, corresponding to a 4-fold increased risk in individuals with muscular dystrophy (HR 4.02, 95% CI 1.73–9.30). Moreover, a nearly 9-fold increased risk of astrocytomas or other gliomas was found among children with muscular dystrophy (HR 8.70, 95% CI 3.57–21.20).

**Table 2:** Results From Cox Proportional Hazards Regression Models on the Association Between Muscular Dystrophy and Specific Cancer Types

	Main analysis		After excluding individuals with DMD and BMD		After excluding individuals with a genetic syndrome	
	N cancer cases muscular dystrophy/N cancer cases comparisons	HR (95% CI)	N cancer cases muscular dystrophy/N cancer cases comparisons	HR (95% CI)	N cancer cases muscular dystrophy/N cancer cases comparisons	HR (95% CI)
Childhood cancer						
Any cancer	8/291	1.35 (0.67–2.73)	5/216	1.13 (0.46–2.75)	7/263	1.29 (0.61–2.75)
CNS tumors	6/74	4.02 (1.73–9.30)	4/54	3.75 (1.34–10.52)	5/65	3.74 (1.49–9.37)
Brain tumors	6/62	4.80 (2.05–11.26)	4/45	4.64 (1.62–13.24)	5/55	4.37 (1.72–11.05)
Astrocytomas and other gliomas	6/33	8.70 (3.57–21.20)	4/25	8.87 (2.94–26.70)	5/28	8.27 (3.11–21.94)
Cancer in adults						
Any cancer	83/4,128	1.04 (0.83–1.29)	76/3,813	1.03 (0.82–1.29)	77/3,944	1.00 (0.80–1.25)
CNS tumor	5/222	1.17 (0.48–2.84)	5/206	1.25 (0.51–3.05)	4/204	1.01 (0.37–2.72)
Brain tumor	5/200	1.31 (0.53–3.18)	5/188	1.39 (0.57–3.38)	4/184	1.12 (0.42–3.03)
Breast cancer	20/817	1.27 (0.81–1.98)	19/779	1.27 (0.80–2.00)	17/778	1.11 (0.69–1.80)
Prostate cancer	5/476	0.53 (0.22–1.28)	5/446	0.56 (0.23–1.36)	5/459	0.55 (0.23–1.34)
Testicular cancer	4/155	1.45 (0.54–3.92)	2/118	0.91 (0.22–3.69)	4/152	1.48 (0.55–4.01)
Bladder	3/91	1.57 (0.50–4.98)	1/84	0.57 (0.08–4.10)	3/89	1.60 (0.51–5.09)
Skin melanoma	9/412	1.14 (0.59–2.21)	8/365	1.15 (0.57–2.32)	8/400	1.04 (0.51–2.09)
Thyroid cancer	3/65	2.46 (0.77–7.86)	3/60	2.71 (0.85–8.71)	3/63	2.54 (0.79–8.12)
Nonthyroid endocrine cancer	6/125	2.35 (1.03–5.34)	6/115	2.58 (1.13–5.87)	6/116	2.54 (1.12–5.79)
Lymphoma	4/165	1.26 (0.47–3.40)	4/152	1.38 (0.51–3.72)	4/162	1.27 (0.47–3.44)
Colon cancer	5/199	1.32 (0.54–3.23)	5/184	1.43 (0.59–3.49)	5/182	1.43 (0.59–3.48)
Pancreatic cancer	4/47	4.33 (1.55–12.11)	4/44	4.71 (1.67–13.22)	4/46	4.43 (1.58–12.40)

Abbreviations: BMD = Becker muscular dystrophy; DMD = Duchenne muscular dystrophy; HR = hazard ratio.

**Table 3:** Results From Cox Proportional Hazards Regression Models on the Association Between Myotonic Dystrophy and Specific Cancer Types

	Main analysis		After excluding individuals with a syndrome	
	N cancer cases myotonic dystrophy/N cancer cases comparisons	HR (95% CI)	N cancer cases myotonic dystrophy/N cancer cases comparisons	HR (95% CI)
Childhood cancer				
Any childhood cancer	9/259	1.70 (0.88–3.32)	8/240	1.63 (0.80–3.29)
Childhood CNS	4/69	2.73 (0.99–7.54)	3/61	2.35 (0.73–7.56)
Childhood brain tumors	4/59	3.23 (1.16–9.01)	3/52	2.73 (0.84–8.90)
Cancer in adults				
Any cancer	149/3,782	2.26 (1.92–2.66)	144/3,615	2.29 (1.94–2.71)
CNS tumor	39/218	9.94 (7.02–14.07)	38/201	10.57 (7.42–15.06)
Brain tumors	37/197	10.44 (7.30–14.95)	36/184	10.91 (7.57–15.72)
Astrocytomas and other gliomas	34/100	17.97 (12.06–26.78)	34/94	19.10 (12.77–28.56)
Breast cancer	10/759	0.78 (0.42–1.46)	10/707	0.84 (0.45–1.57)
Prostate cancer	6/406	1.05 (0.47–2.37)	6/401	1.07 (0.48–2.42)
Bladder cancer	3/85	2.20 (0.69–7.03)	3/83	2.28 (0.71–7.27)
Skin melanoma	9/370	1.28 (0.66–2.48)	9/356	1.33 (0.68–2.58)
Thyroid cancer	6/76	3.92 (1.70–9.03)	6/71	4.21 (1.82–9.75)
Nonthyroid endocrine cancer	17/108	7.49 (4.47–12.56)	16/106	7.16 (4.21–12.17)
Colon cancer	5/178	1.61 (0.66–3.93)	4/169	1.36 (0.50–3.69)
Cancer of the rectum	4/118	2.16 (0.79–5.89)	4/115	2.32 (0.82–6.10)
Endometrial cancer	10/70	8.32 (4.22–16.40)	9/70	7.46 (3.67–15.15)
Ovarian cancer	5/71	4.00 (1.60–10.01)	5/67	4.14 (1.65–10.35)
Kidney cancer	3/72	2.42 (0.76–7.36)	3/69	2.54 (0.79–8.15)
Nonmelanoma skin cancer	5/95	3.27 (1.32–8.13)	5/89	3.52 (1.41–8.77)

Abbreviation: HR = hazard ratio.

There were 83 adults with muscular dystrophy and a cancer diagnosis, with the most common being breast cancer (n = 30) and skin melanoma (n = 9). In adults with muscular dystrophy, we found an increased risk of nonthyroid endocrine cancer (HR 2.35, 95% CI 1.03–5.34) and pancreatic cancer (HR 4.33, 95% CI 1.55–12.11). Despite the increased risk in childhood, adults with muscular dystrophy were not at an increased risk of CNS tumors (HR 1.17, 95% CI 0.48–2.84). To rule out that the observed cancer risk increase was explained by DMD and BMD alone, we performed sensitivity analyses excluding patients with these diagnoses in the NPR. Similar results were seen after exclusion (Table 2).

In individuals with myotonic dystrophy, an increased risk of childhood brain tumors (HR 3.23, 95% CI 1.19–9.10) was observed, although there were only 4 children with myotonic dystrophy and this cancer type. There were 149 adults with myotonic dystrophy who had a cancer diagnosis, with the most common being CNS tumors (n = 39), nonthyroid endocrine cancer (n = 17), and breast and endometrial cancer (n = 10). Individuals with myotonic dystrophy presented a 2-fold increased risk of cancer in adulthood (Table 3), likely driven by the particularly increased risk of astrocytomas and other gliomas (HR 17.97, 95% CI 12.06–26.78). These patients also had a nearly 4-fold increased risk of thyroid, ovarian, and nonmelanoma skin cancer and an 8-fold increased risk of nonthyroid endocrine and endometrial malignancies (Table 3). Similar results were observed in all analyses after excluding individuals with other genetic syndromes (Tables 2 and 3).

In our sex-stratified analyses, we found that the increased risk of childhood brain tumors in individuals with muscular dystrophy and myotonic dystrophy was confined to boys (Tables 4 and 5). Regarding adult cancer among patients with muscular dystrophy, an increased risk of nonthyroid endocrine cancer was observed only in women, while a 7-fold increased risk of pancreatic cancer only in men (Table 4). These differences remained significant after excluding X-linked disorders, namely individuals diagnosed with DMD and BMD (eTable 1). Both men and women with myotonic dystrophy presented an increased risk of astrocytomas and other gliomas (HR 21.83, 95% CI 12.61–37.78; HR 15.35, 95% CI 8.05–29.27, respectively), while an increased risk of thyroid, nonthyroid endocrine, and nonmelanoma skin cancer was observed only in women (Table 5).

**Table 4:** Results From Cox Proportional Hazards Regression Models on the Association Between Muscular Dystrophy and Specific Cancer Types Stratified by Sex

	Men		Women	
	N cancer muscular dystrophy/N cancer comparisons	HR (95% CI)	N cancer muscular dystrophy/N cancer comparisons	HR (95% CI)
Childhood cancer				
Any childhood cancer	6/194	1.55 (0.68–3.51)	2/97	—
Childhood CNS	5/44	6.14 (2.39–15.74)	1/30	—
Childhood brain tumor	5/38	7.68 (2.94–20.06)	1/24	—
Astrocytomas and other gliomas	5/20	13.74 (4.99–37.83)	1/13	—
Cancer in adults				
Any cancer	32/1,990	0.85 (0.60–1.21)	51/2,138	1.20 (0.91–1.59)
CNS tumor	0/121	—	5/101	2.56 (1.04–6.31)
Brain tumor	0/108	—	5/92	2.79 (1.13–6.89)
Bladder	3/69	2.11 (0.66–6.72)	0/22	—
Skin melanoma	3/195	0.87 (0.28–2.72)	6/217	1.34 (0.60–3.04)
Thyroid cancer	1/27	—	2/38	—
Nonthyroid endocrine cancer	2/57	—	4/68	2.83 (1.03–7.78)
Lymphoma	1/101	—	3/64	2.45 (0.77–7.83)
Colon cancer	4/115	1.87 (0.68–5.08)	1/84	—
Pancreatic cancer	3/25	7.06 (2.08–23.96)	1/22	—

Abbreviation: HR = hazard ratio.

**Table 5:** Results From Cox Proportional Hazards Regression Models on the Association Between Myotonic Dystrophy and Specific Cancer Types Stratified by Sex

	Men		Women	
	N cancer myotonic dystrophy/N cancer comparisons	HR (95% CI)	N cancer myotonic dystrophy/N cancer comparisons	HR (95% CI)
Childhood cancer				
Any childhood cancer	9/131	3.43 (1.74–3.76)	0/128	—
Childhood CNS	4/31	5.76 (2.01–16.53)	0/38	—
Childhood brain tumors	4/24	6.88 (2.30–20.53)	0/35	—
Cancer in adults				
Any cancer	58/1,672	2.03 (1.56–2.64)	91/2,110	2.42 (1.96–2.99)
CNS tumor	23/98	12.91 (8.06–20.68)	16/120	7.69 (4.51–13.12)
Brain tumors	22/93	13.04 (8.04–21.14)	15/104	8.41 (4.82–14.66)
Astrocytomas and other gliomas	21/53	21.83 (12.61–37.78)	13/47	15.35 (8.05–29.27)
Bladder cancer	1/61	—	2/24	—
Skin melanoma	5/157	1.71 (0.70–4.18)	4/213	0.98 (0.36–2.64)
Thyroid cancer	1/23	—	5/53	4.83 (1.90–12.28)
Nonthyroid endocrine cancer	3/48	2.88 (0.88–9.40)	14/60	11.77 (6.52–21.59)
Colon cancer	2/90	—	3/88	2.21 (0.69–7.12)
Cancer of the rectum	2/70	—	2/48	—
Kidney cancer	3/51	2.89 (0.88–9.51)	0/21	—
Nonmelanoma skin cancer	1/60	—	4/35	9.38 (3.10–28.37)

Abbreviation: HR = hazard ratio.

## Discussion

In this large Swedish register-based study, we found that patients with muscular dystrophy are at an increased risk of childhood CNS tumors, particularly astrocytomas and other gliomas, while adults present a higher risk of nonthyroid endocrine cancer and pancreatic cancer. To the best of our knowledge, this is the first study reporting specific cancer associations for individuals with muscular dystrophy. We also observed that patients with myotonic dystrophy are at an overall increased risk of cancer during childhood and adulthood. This risk is likely driven by CNS tumors during childhood, and multiple malignancies in adulthood, including astrocytomas and other gliomas, thyroid, ovarian, endocrine, endometrial, and nonmelanoma skin cancer.

In our literature review of published reports and epidemiologic studies of muscular dystrophy or myotonic dystrophy and cancer (eMethods and eTable 2), we observed an overrepresentation of sarcomas in patients with cancer and DMD (9/23 [39%], as compared with a sarcoma proportion of 0.7% among all cancers, according to the Surveillance, Epidemiology, and End Results^29^). In our epidemiologic study however, only 1 sarcoma in a patient with myotonic dystrophy was observed, suggesting that the high number of reported sarcoma cases in DMD may be a publication bias. Possibly, these presentations were interesting because of previous studies confirming an increased risk of sarcoma in mouse models of various muscular dystrophies.^10-13,30,31^ Additional epidemiologic studies are warranted to confirm that patients with DMD are not at a higher risk of sarcomas. Of note, more than 60% of patients with muscular dystrophy in our study were male. This is to be expected as DMD and BMD are X-linked disorders.

Previous epidemiologic studies have found an overall increased cancer risk in patients with myotonic dystrophy, as well as a high risk of brain tumors,^14,15,20^ thyroid,^16,19,20^ endometrial,^14,17,20^ ovarian,^14,20^ and skin cancer^18,19^ in this patient group—results that were confirmed in our study. However, our results did not confirm the reported increased risk of lymphoma,^17^ colon,^14,17^ testicular,^16,17^ and prostate^16^ cancer. In addition, we found that adults with myotonic dystrophy are at an increased risk of nonthyroid endocrine tumors.

The detection of cancer predisposition in congenital syndromes may have important clinical implications because surveillance and early detection of cancer may result in improved outcomes. In addition, altered treatment strategies may be offered for some diagnoses. Confirming that patients are not at a higher risk of cancer—for instance, not finding the suspected increased risk of sarcoma in muscular dystrophy—also conveys useful information for clinical care. However, this study is based on *ICD* codes and a relatively small Swedish cohort without molecularly confirmed subtypes of muscular dystrophies and myotonic dystrophy. Therefore, further studies confirming and delineating the increased cancer risks are needed before altered cancer surveillance regimens can be considered.

Multiple studies have also reported sex differences in the cancer risk in individuals with myotonic dystrophy, indicating that female patients are at a higher risk of malignancies.^20,32-35^ In this study, we found that both men and women with myotonic dystrophy had a 2-fold increased risk of cancer during adulthood, including an increased risk of astrocytoma and other gliomas. However, a specific risk of thyroid, nonthyroid, and nonmelanoma skin cancer was observed only in women. Similarly, we observed sex differences in the cancer risk spectrum of individuals with muscular dystrophy. An increased risk of nonthyroid endocrine cancer was observed only in women, while that of pancreatic cancer was found only in men. Finally, only boys with muscular dystrophy and myotonic dystrophy were at an increased risk of childhood brain tumors. All in all, these results suggest that the cancer risk spectrum in muscular dystrophy and myotonic dystrophy is sex-dependent.

There are multiple hypotheses that could explain these differences, such as global gene expression changes and epigenetic mechanisms. However, in muscular dystrophy these differences could also be explained by the fact that the number of women is nearly half of the number of men because Duchenne and Becker muscular dystrophies are X-linked disorders. However, the observed sex differences in the cancer risk spectrum remained significant after excluding patients diagnosed with Duchenne and Becker muscular dystrophies.

Remarkably, we observed a similar cancer risk spectrum in patients with muscular dystrophy and myotonic dystrophy, including nonthyroid endocrine tumors and CNS tumors, particularly astrocytomas and gliomas. In addition, we found an increased risk of pancreatic cancer in adults with muscular dystrophy, although this finding was only based on 4 cases. No increased risk of pancreatic cancer was found among adults with myotonic dystrophy, despite this association being reported in a previous Nordic study.^14^ Furthermore, according to our sensitivity analysis excluding patients with DMD and BMD, the reported cancer risk increase in muscular dystrophy is not explained by cancer in patients with *DMD*-associated diseases exclusively.

One of the main limitations of this study is that muscular dystrophy and myotonic dystrophy are rare diseases. Therefore, there was not enough statistical power to study each muscular dystrophy type independently. As the *ICD* code for muscular dystrophy covers multiple subtypes, it is possible that different muscular dystrophies are associated with distinct cancer risks. It is therefore likely that grouping them may result in missed associations between cancer and specific muscular dystrophies. Similarly, we cannot exclude the possibility that the increased risk for some cancer types in muscular dystrophy presented in this study are true only for certain muscular dystrophy subtypes.

Moreover, several of our associations are based on a limited number of patients with muscular dystrophy and myotonic dystrophy. Therefore, we cannot rule out the possibility that some of our findings, especially the ones regarding the increased risk of nonthyroid endocrine tumors and pancreatic cancer in muscular dystrophy or results from sex-stratified analyses, could be due to chance. Similarly, to the best of our knowledge, this is the first study reporting specific cancer associations for individuals with muscular dystrophy and an increased risk of nonthyroid endocrine tumors among individuals with myotonic dystrophy. Hence, we cannot discard that these results were found by chance because of the several associations evaluated in the study. Therefore, we believe that these results should be interpreted with caution and will need to be replicated on independent cohorts.

A second limitation of our study is the clinical classification of myotonic dystrophy that is, as a type of muscular dystrophy. This resulted in a dual diagnosis of muscular dystrophy and myotonic dystrophy for a group of individuals (18%) in the NPR. We separated the diagnoses to the best of our ability (see methods section). However, a misclassification of the disease subgroup (i.e., muscular dystrophy vs myotonic dystrophy) cannot be excluded. Similarly, codes for muscular dystrophy and myotonic dystrophy were available in *ICD-9*, but not for BMD and DMD. Therefore, some individuals diagnosed with muscular dystrophy in *ICD-9*, have DMD or BMD. Given the previously discussed finding of an increased risk of CNS tumors and nonthyroid endocrine cancer in patients with muscular dystrophy and myotonic dystrophy, additional studies are needed to clarify whether the cancer risk spectrum overlaps or whether these findings are caused by disease misdiagnosis.

Another limitation is that some individuals with muscular dystrophy or myotonic dystrophy might not have had a diagnosis reported to the patient register, for instance in young individuals, if the diagnosis was not yet made. This is suggested by the fact that the number of patients with these conditions was lower in more recent birth years. However, the likelihood of including them as matched comparisons is quite low. Therefore, we believe that this will have no or low impact on the results of our study. Another concern is that, as we include individuals born 1950–2017, patients with myotonic dystrophy or muscular dystrophy in the recent birth cohorts might have a different disease profile than individuals in older birth cohorts, including for instance, fewer dystrophies with later onset and more congenital dystrophies thanks to the possibility of genetic testing. Different disease profiles could have distinct cancer risk associations; however, we did not have enough statistical power to perform analyses stratified by birth cohort. It is important to notice that this limitation is less of a concern in the results of the cancer risk in adults, as we include only individuals born 1950–1997.

In this population-based study, we show an increased risk of childhood CNS tumors, particularly astrocytomas and other gliomas, as well as nonthyroid endocrine and pancreatic cancer in adults with muscular dystrophy. We also observed an overall cancer risk increase in patients with myotonic dystrophy. Specifically, high risk of astrocytomas and other gliomas in children and thyroid, ovarian, endocrine, endometrial, and nonmelanoma skin cancer in adults. The results from large epidemiologic studies such as ours are important for understanding the cancer predisposition spectrum in patients with rare diseases, including muscular dystrophy and myotonic dystrophy. An increased knowledge on cancer risk in rare diseases will, in turn, facilitate early cancer diagnosis and, when available, initiation of surveillance programs in the clinic. Further investigations are needed to elucidate the reasons for the detected higher cancer risks and sex differences. Future epidemiologic studies on larger cohorts, with genetically confirmed subtypes of muscular dystrophies and myotonic dystrophies are warranted, to confirm and delineate cancer risks for different subtypes, to improve cancer surveillance and clinical care in these patient groups.

## Appendix Authors

**Table:**

Name	Location	Contribution
Carolina Maya-González	Department of Molecular Medicine and Surgery, Center for Molecular Medicine, Karolinska Institutet, Stockholm, Sweden	Drafting/revision of the manuscript for content; major role in the acquisition of data; analysis or interpretation of data
Giorgio Tettamanti, PhD	Department of Molecular Medicine and Surgery, Center for Molecular Medicine, and Unit of Epidemiology, Institute of Environmental Medicine, Karolinska Institutet, Stockholm, Sweden	Drafting/revision of the manuscript for content; major role in the acquisition of data; analysis or interpretation of data
Fulya Taylan, PhD	Department of Molecular Medicine and Surgery, Center for Molecular Medicine, Karolinska Institutet; Department of Clinical Genetics and Genomics, Karolinska University Hospital, Stockholm, Sweden	Drafting/revision of the manuscript for content; major role in the acquisition of data; analysis or interpretation of data
Anna Skarin Nordenvall, MD, PhD	Department of Molecular Medicine and Surgery, Center for Molecular Medicine, Karolinska Institutet; Department of Radiology, Karolinska University Hospital, Stockholm, Sweden	Study concept or design; analysis or interpretation of data
Thomas Sejersen, MD, PhD	Department of Child Neurology, Astrid Lindgren Children's Hospital, Karolinska University Hospital; Department of Women's and Children's Health, Karolinska Institutet, Stockholm, Sweden	Drafting/revision of the manuscript for content, including medical writing for content
Ann Nordgren, MD, PhD	Department of Molecular Medicine and Surgery, Center for Molecular Medicine, Karolinska Institutet; Department of Clinical Genetics and Genomics, Karolinska University Hospital, Stockholm; Department of Clinical Genetics and Genomics, Sahlgrenska University Hospital, Gothenburg; Institute of Biomedicine, Department of Laboratory Medicine, University of Gothenburg, Sweden	Drafting/revision of the manuscript for content, including medical writing for content; major role in the acquisition of data; study concept or design; analysis or interpretation of data

## Glossary

BMD: Becker muscular dystrophy
DMD: Duchenne muscular dystrophy
HR: hazard ratio
*ICD-9*: *International Classification of Diseases, Ninth Revision*
*ICD-10*: *International Classification of Diseases, 10th Revision*
NPR: National Patient Register
